# Cost Comparisons of Physician-Ordered Versus Direct-to-Consumer Laboratory Testing

**DOI:** 10.7759/cureus.74173

**Published:** 2024-11-21

**Authors:** Trevor Lin, Curtis E Margo

**Affiliations:** 1 Department of Ophthalmology, Morsani College of Medicine, University of South Florida, Tampa, USA; 2 Department of Pathology and Cell Biology, Morsani College of Medicine, University of South Florida, Tampa, USA

**Keywords:** laboratory charges, laboratory costs, laboratory tests, patient education, uninsured patients

## Abstract

Objectives

To determine if direct-to-consumer (DTC) laboratory testing is less expensive for patients, as is generally advertised, and whether it has any role in managing uninsured or underinsured patients.

Methods

The costs of six commonly ordered laboratory tests were obtained through two major DTC laboratories and compared with 42 physician-ordered, hospital-based laboratories in Florida. The costs of DTC tests were also compared to concurrent reimbursements from Medicare and Medicaid.

Results

The costs of DTC tests were generally lower than the mean insurance-negotiated hospital-based prices and much less costly than physician-ordered tests for patients without insurance. Charges were greater than reimbursements from Medicaid and Medicare. The inter-hospital variation in the pricing of laboratory tests was remarkably wide for both insured and uninsured patients.

Conclusions

Charges to insured patients for physician-ordered laboratory tests tend to be higher than those offered directly to consumers, but out-of-pocket costs will vary depending on deductibles and co-pays. DTC costs would be substantially less for uninsured patients when ordered by a patient under the supervision of a physician.

## Introduction

Clinical laboratories are essential parts of the healthcare system, and the results of laboratory tests play critical roles in the diagnosis and management of many human maladies [1]. Reimbursements for these services in the United States come from three sources: (1) governments, (2) third-party payors, and (3) patients. Until recently, these laboratory services have been selected and ordered by a qualified medical practitioner (e.g., medical doctor and doctor of osteopathy), who determined what tests were necessary and then provided patients with expert interpretation and explanation of the results. Within the last few years, however, several certified laboratories have been offering select laboratory tests directly to the public.

Direct-to-consumer (DTC) laboratory testing is the latest alternative to the traditional delivery of physician-centered medical care. Practical and ethical concerns regarding DTC laboratory testing have been raised in the medical literature [2-4], but its ultimate success may be driven by market forces. The ease of access and the relatively lower costs of DTC testing compared to services through physician providers are key marketing advantages. Since it is unclear how the costs of DTC laboratory testing compare to charges through traditional routes of delivery, we conducted a price comparison between physician-ordered and consumer-ordered tests for select laboratory tests in Florida.

## Materials and methods

Study design

This study presents the direct comparison of the costs for the same outpatient laboratory tests when ordered by a physician versus when ordered by a patient (i.e., DTC price).

Data collection

The costs of six common laboratory tests, when ordered by physicians through Florida hospitals with 400 or more staffed beds, were accessed online [5]. This was made possible by the Centers for Medicare & Medicaid Services (CMS) Hospital Price Transparency rules [6]. Pricing data from each hospital's machine-readable file for each test included the gross charges, cash-discounted prices, insurance plan-specific negotiated prices, and minimum and maximum negotiated prices. DTC prices in Florida were obtained online from Quest Diagnostics and Labcorp [7,8]. The six tests were identified using the Current Procedural Terminology (CPT): complete blood count (CBC) (CPT 85025 (85027 for HCA-owned hospitals)); hemoglobin A1c (CPT 83036); vitamin D (CPT 82306); complete metabolic panel (CPT 80053); lipid panel (CPT 80061); and prostate-specific antigen (PSA) (CPT 84153). For each laboratory test and hospital, the cost to an insured patient was determined by calculating the average of all insurance-negotiated charges.

Florida Medicaid and Medicare reimbursements for 2024 were obtained online from the Agency for Health Care Administration and the CMS, respectively [9,10].

Statistical analysis

Descriptive statistical analyses (mean, standard deviation, median, and range) were performed using Microsoft Excel Analysis ToolPak (Microsoft Corp., Redmond, WA, United States).

## Results

**Figure 1:**
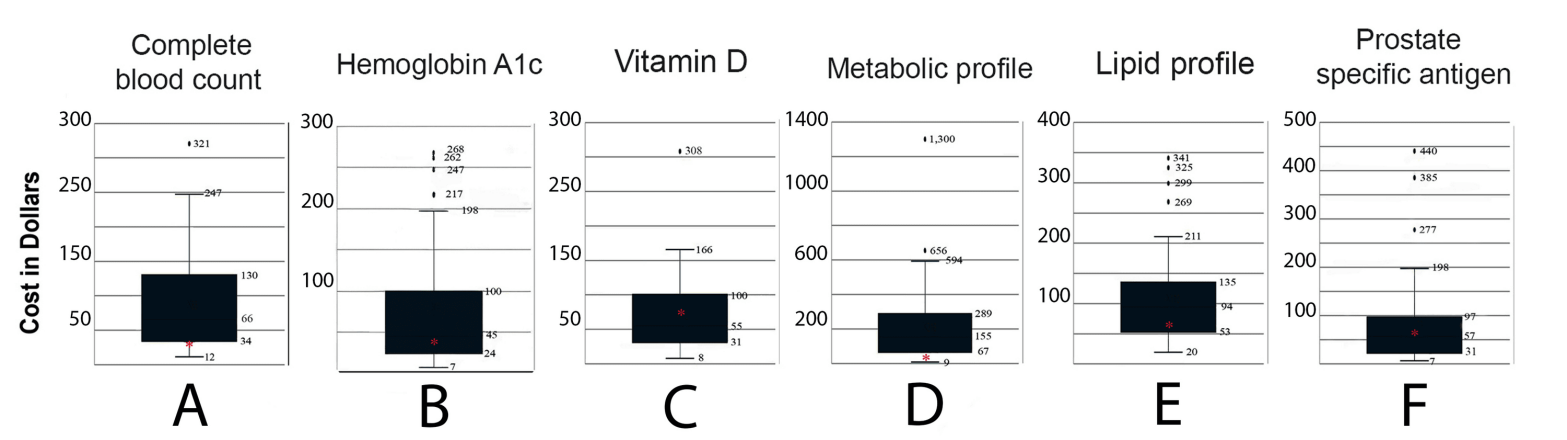
Box plot with whiskers Graphs of hospital-negotiated prices (hospital insurance prices) showing first and third quartile ranges and minimum and maximum charges for six commonly ordered tests: complete blood count (A), hemoglobin A1c (B), vitamin D (C), metabolic profile (D), lipid profile (E), prostate-specific antigen (F). The asterisk (*) represents the mean direct-to-consumer charge for each test.

Charges for laboratory tests were accessible for 42 of 43 eligible hospitals. The average charge for the six laboratory tests was greatest when ordered by a physician as an uninsured hospital outpatient, followed in descending order by cash discount price, hospital insurance-negotiated price, and DTC price (Table 1). The difference was often substantial. A 12-fold difference in cost was noted between the mean outpatient-ordered CBC for an uninsured patient and the mean CBC ordered directly by the patient (e.g., $401 vs. $32). The range in charges among hospitals was also sizable for all physician-ordered charges (uninsured, cash, and insurance-negotiated) (Table 1). The cash price for a metabolic profile, for instance, ranged from $13 to $2,943. The variation among insurance-negotiated prices was not as large but remained consequential (Figure 1). A PSA, for example, ranged from $7 to $440. Reimbursements through Florida Medicaid were substantially lower than the DTC prices, while those for patients enrolled in Medicare were, on average, 45% higher (Table 1). 

**Table 1:** Comparative charges* for physician-ordered versus direct-to-consumer laboratory tests The asterisk (*) represents charges rounded off in dollars; dagger (†), hospital-negotiated prices; double dagger (‡), Quest Diagnostics and Labcorp; pi (π), professional and technical fees.

Laboratory test	Hospital outpatient charge ($)* (n = 42)	Hospital cash price ($) (n = 42)	Hospital insurance price^†^ ($) (n = 42)	Direct-to-consumer cost ($) (n = 2^‡^)	2024 Florida Medicaid/Medicare reimbursement^π^
Complete blood count					$5.26/$7.77
Mean ± standard deviation (SD)	401.00 ± 322.00	296.00 ± 355.00	88.00 ± 70.00	32.00 ± 4.00	
Median (min, max)	318.00 (23.00, 1381.00)	141.00 (4.00, 1381.00)	66.00 (12.00, 321.00)	32.00 (29.00, 35.00)	
Hemoglobin A1c					$6.26/$9.71
Mean ± SD	250.00 ± 220.00	151.00 ± 180.00	81.00 ± 79.00	42.00 ± 4.00	
Median, (Min, Max)	165.00, (9.00, 849.00)	74.00, (9.00, 849.00)	45.00, (7.00, 269.00)	42.00, (39.00, 45.00)	
Vitamin D					$19.75/$29.60
Mean ± SD	173.00 ± 148.00	104.00 ± 130.00	72.00 ± 56.00	87.00 ± 17.00	
Median (min, max)	134.00 (8.00, 710.00)	61.00 (8.00, 710.00)	55.00 (8.00, 308.00)	87.00 (75.00, 99.00)	
Metabolic profile					$7.32/$10.56
Mean ± SD	957.00 ± 834.00	663.00 ± 831.00	218.00 ± 238.00	52.00 ± 4.00	
Median (min, max)	873.00 (32.00, 2943.00)	291.00 (13.00, 2943.00)	155.00 (9.00, 1300.00)	52.00 (49.00, 55.00)	
Lipid profile					$8.06/$13.39
Mean ± SD	558.00 ± 511.00	412.00 ± 547.00	110.00 ± 81.00	62.00 ± 4.00	
Median (min, max)	419.00 (33.00, 2072.00)	171.00 (6.00, 2072.00)	94.00 (20.00, 341.00)	62.00 (59.00, 65.00)	
Prostate-specific antigen					$14.16/$18.39
Mean ± SD	276.00 ± 298.00	170.00 ± 255.00	83.00 ± 95.00	72.00 ± 4.00	
Median (min, max)	139.00 (6.00, 1050.00)	64.00 (6.00, 1050.00)	57.00 (7.00, 440.00)	72.00 (69.00, 75.00)	

## Discussion

The interest in conducting this study arose after an insured patient enrolled in a highly rated Medicare Advantage Plan was unable to obtain an HbA1c test during an annual examination because it was not considered medically justified for a routine check-up (only fasting blood glucose was authorized) [11]. The HbA1c could have been ordered separately (i.e., physician-ordered) and the study performed by the hospital outpatient laboratory, but it would have to be paid for out-of-pocket, at $245.00. The same test, however, was instead performed without a physician order (i.e., DTC) and through a Clinical Laboratory Improvement Amendment (CLIA)-certified laboratory at a cost of only $39.00. The cost saving on this single laboratory test spurred interest in the magnitude of savings that might be realized for other commonly ordered studies. The disparities in charges also raised questions as to why such a large variation in price occurred in clinical laboratories serving the same community in the first place.

Background on laboratory pricing

How the cost of individual laboratory tests is determined is arcane and not at all transparent [12]. The roles that governments, third-party payors, and individual laboratories play in determining financial reimbursements are intertwined. The process has become increasingly complex since the 1980s when CMS separated the mechanisms for payment of medical services based on whether the laboratory test was ordered for an inpatient or outpatient [13]. In 1982, Congress created the prospective payment system to help contain medical expenditures, and in so doing developed diagnostic-related groups (DRGs) (or idealized homogenous diagnostic categories) to facilitate payments for inpatient services. Each DRG established a reimbursement rate for a specific diagnostic group of inpatients. In so doing, laboratory services were “bundled” into a single fee for that group, no matter how few or how many tests were done. Laboratories were thus considered “cost centers,” and the business model favored testing parsimony. This method of prospective payment resulted in a shift in many traditional inpatient services to outpatient and forced Medicare to deal with a growing volume of outpatient laboratory testing. In 1984, Medicare unveiled the Medicare Clinical Laboratory Fee Schedule, which is still used to establish reimbursements for most clinical laboratory services [14]. The formula for determining Medicare reimbursements is adjusted for 56 geographic jurisdictions and is periodically updated.

Although Medicare is the single largest payor for laboratory services, private insurance groups, through indemnity and managed care plans, are collectively larger. In terms of how laboratory reimbursement fees are determined, CMS sets those rates based on the fees negotiated between private insurers and private laboratories. The methodology also relies on whether the ordering CPT laboratory code is medically justified. To accomplish this, the ordering physician must document medical necessity using an appropriate International Classification of Disease, Tenth Revision (ICD-10) code (or in the past, earlier Revisions). Failure to document medical necessity through ICD coding will result in denial of payment [14].

Medicare determines fee schedules based on retrospective billing data from laboratories. Clinical laboratories submit average rates for what private payors had paid for services over the previous three years [10]. These three-year averages are adjusted for geographic location, inflation, etc., and finally, a clinical laboratory fee schedule is developed. As one can see, it is to a clinical laboratory’s advantage to negotiate as high a reimbursement fee from insurance carriers as possible since it ultimately affects the Clinical Laboratory Fee Schedule used by the CMS. To gain some leverage over insurance carriers, clinical laboratories are motivated to set high retail prices so that they have room to negotiate with insurers. The high retail fees used in negotiations, however, are those that uninsured patients will have to pay.

Critique

The six commonly ordered laboratory tests examined in this study revealed remarkable variations in charges for the same test depending on how it was ordered. Studies ordered by physicians were more expensive than those purchased directly by consumers, although some insurance-negotiated hospital prices tended to be competitive with DTC costs. The ranges in insurance-negotiated prices, however, were unaccountably broad. The more than 140-fold difference in charges for a metabolic profile, for instance, cannot be understood in terms of the cost incurred by laboratories for materials and labor to perform the test. The large variation of DTC pricing has been documented in another study looking at seven frequently ordered laboratory tests [15]. The authors of that study also found the variation inexplicable.

The subject of inflated physician-ordered laboratory charges has been discussed in the news media, often in the context of “sticker shock” or price gouging [16,17]. These cases often represent patients who are insured but not aware of high deductibles or co-pays or do not know that their coverage could be denied if the order lacked appropriate coding for medical necessity. The medical literature dealing with DTC laboratory testing has focused on quality of care and professional ethics [2-4], arguing that patients may not understand the medical implications of their test results or may misinterpret them. While these arguments have merit, the public may frame the choice between a physician- and a consumer-ordered testing from a solely financial perspective.

Physicians caring for uninsured patients may want to direct patients who need a prescribed set of laboratory studies to DTC laboratories rather than have them take physician-signed order sheets to hospital-run laboratories. A 2023 survey found that a large number of laboratory tests are now available in the United States through a variety of companies [18].

What is often overlooked in the discussion over the pricing of laboratory tests is that most laboratories also participate in state Medicaid, whose reimbursements (last column in Table 1) are exceptionally low. States like Florida have the authority to further adjust the reimbursement rates set by the CMS downward. Such low reimbursement schedules for Medicaid likely affect how laboratories negotiate with insurance companies in the future, since CMS fees for both Medicare and Medicaid are based on formulas that use weighted median private payor rates [19]. Given the complexity of determining the “real” cost of performing individual laboratory tests (e.g., the sum of direct and indirect costs, operational costs, and costs of quality control), laboratories may lose money on some Medicaid fee schedules. Such financial hardships were experienced for inpatient laboratory services after the method of DRG reimbursements was put in place in the 1980s [20]. Laboratories that can minimize their exposure to patients with Medicaid have a financial advantage over those that decide to serve them.

The closeness in DTC prices offered by the laboratories in this study is consistent with consumer-driven competition. Given the economic burden that medical expenses in the United States place on many individuals and families [21], physicians may want to consider supervising select patients in their use of DTC laboratories for needed studies rather than hospital-based laboratories when financial hardships may prevent them from even getting tested. 

Limitations

The limitations of this study are the small sample of laboratory tests, although the six selected are some of the most commonly ordered. Although just two DTC medical laboratories were appraised, they are the two dominant laboratories in the state with widely available customer centers near the hospital-operated laboratories in this study. Fee schedules as discussed in this paper also have little relevance to federal laboratories like those operating through the Veterans Administration.

## Conclusions

DTC costs for six commonly ordered laboratory tests are generally lower than insurance-negotiated, physician-ordered studies and are substantially less expensive for those without insurance. Insurance-negotiated prices for tests also vary widely from averages within the same hospital laboratory. These wide variations in prices are likely a reflection of how laboratory administrators cope with the formulas used by states and CMS to reimburse laboratories for persons covered by Medicaid and Medicare. 
